# Mismatch-Enhanced Specific PCR (MES-PCR): A Rapid and Cost-Effective Method for Screening CRISPR/Cas9-Induced Mutations

**DOI:** 10.3390/biology15131089

**Published:** 2026-07-07

**Authors:** Peng Tian, Bengang Yao, Wenjing Lin, Maoting Yuan, Shuran Li, Yuzhu Qin, Shuang Chen, Tao Lai, Zhenbiao Yang, Wenwei Lin, Xiang Zhou

**Affiliations:** 1 Fujian Provincial Key Laboratory of Haixia Applied Plant Systems Biology, College of Life Science, Fujian Agriculture and Forestry University, Fuzhou 350002, China; tianp17@foxmail.com (P.T.); bengangyao@126.com (B.Y.); wenjinglin2026@outlook.com (W.L.); maot2023@163.com (M.Y.); lsr202606@163.com (S.L.); yuzhuqin02@163.com (Y.Q.); shuangchen1026@126.com (S.C.); 2 Horticultural Plant Biology and Metabolomics Center, Haixia Institute of Science and Technology, Fujian Agriculture and Forestry University, Fuzhou 350002, China; yangzhenbiao@suat-sz.edu.cn; 3 State Key Laboratory of Quantitative Synthetic Biology, Shenzhen Institute of Synthetic Biology, Shenzhen Institutes of Advanced Technology, Chinese Academy of Sciences, Shenzhen 518055, China; laitao4396@163.com

**Keywords:** ACT-PCR, MES-PCR, mutation, CRISPR/Cas9, genotyping

## Abstract

CRISPR-associated protein 9 (Cas9)-mediated editing generates numerous mutations. Existing detection methods, such as ACT-PCR, T7EI endonuclease cleavage, HRM analysis, and high-throughput sequencing, often require stringent conditions, expensive reagents, or specialized instruments. Here, we introduce Mismatch-Enhanced Specific PCR (MES-PCR), a method that offers sensitivity for detecting mutations under non-stringent experimental conditions. Combined with quantitative PCR (MES-qPCR), it facilitates the calculation of sgRNA efficiency and enables screening for heterozygous mutations. We validated this method in soybean (*Glycine max* (L.) Merr.) and *Arabidopsis thaliana*, confirming its practicality. This approach significantly enhances the efficiency and reduces the cost of mutation screening, presenting a powerful tool to accelerate precision breeding and functional genomics research in crops.

## Simple Summary

The CRISPR/Cas9 system has been widely used for genome editing, enabling the efficient generation of mutations in target genes. However, current methods for identifying these mutations, such as ACT-PCR, T7 endonuclease I (T7EI) cleavage, high-resolution melting (HRM) analysis, and high-throughput sequencing, suffer from limitations including stringent reaction conditions, high-cost reagents or instruments, operational complexity, or insufficient sensitivity for heterozygous and single-nucleotide variants. To address this, we developed a novel PCR-based method termed Mismatch-Enhanced Specific PCR (MES-PCR) for the rapid identification of CRISPR/Cas9-induced mutations. This method employs primers with artificially introduced mismatched bases, enabling high-efficiency discrimination between wild-type and mutant alleles without precise annealing temperature control. It is suitable for detecting both known and unknown mutations, including those distant from the PAM site. When coupled with quantitative PCR, the method can calculate the editing efficiency of sgRNAs and screen heterozygous mutants. We validated MES-PCR in soybean and *Arabidopsis thaliana* gene-edited materials, demonstrating that it has high accuracy in preliminary screening while being significantly simpler and more cost effective. Thus MES-PCR provides an accessible and high-throughput-compatible strategy for screening genome-edited mutants and for the preliminary evaluation of sgRNA efficiency in plant functional genomics and breeding applications.

## 1. Introduction

Gene editing has become a powerful and widely adopted tool in biological research and breeding, as it allows for the rapid introduction of targeted mutations. Following DNA cleavage by engineered nucleases, repair through non-homologous end joining (NHEJ) results in mutations. The subsequent screening and identification of edited alleles constitutes an essential step in the workflow. Current methods for detecting these mutations are broadly categorized into PCR-based techniques and sequencing-based technologies. Common PCR-based approaches include Allele-Specific PCR (ARMS-PCR) [1], Annealing Control Temperature PCR (ACT-PCR) [2], PCR/restriction enzyme (RE) analysis [3], PCR/ribonucleoprotein (RNP) assay [4], T7EI mismatch cleavage [5], and High-Resolution Melting Analysis (HRMA) [6]. For sequencing-based technologies, Sanger sequencing, next-generation sequencing (NGS, e.g., Hi-TOM [7]), and nanopore sequencing [8] are primarily employed (Table 1).

Collectively, these limitations highlight the demand for a detection method that is simpler, more accurate, and more cost effective. To bridge this gap, we developed MES-PCR, which integrates the allele-specificity principle of ARMS-PCR with the operational simplicity of ACT-PCR, while effectively overcoming their respective drawbacks. Specifically, MES-PCR incorporates artificially mismatched bases into the primers, which minimizes primer tolerance to sequence mismatches at the 3′ end. This design eliminates the need for precise annealing temperature optimization (i.e., gradient PCR) and enhances the method’s general applicability under standard PCR conditions. Meanwhile, MES-qPCR enables not only the rapid identification of both known and unknown mutations but also the quantitative assessment of sgRNA editing efficiency and reliable discrimination between heterozygous and homozygous mutants, as successfully validated in both *Arabidopsis thaliana* and soybean in this study. Therefore, MES-PCR provides a robust, accessible, and economical tool for high-throughput genotyping in plant genome editing and breeding programs.

## 2. Materials and Methods

### 2.1. Plant Materials

All mutant materials involved in the experiments were generated by our laboratory.

### 2.2. DNA Extraction

Grind 100 mg of plant leaf (root tissues were used when evaluating the sgRNA editing efficiency in soybean) into a fine powder in liquid nitrogen. Quickly add 500 μL of CTAB extraction buffer (2% CTAB, 1.21% Tris, 1.86% EDTA, 8.2% NaCl, pH adjusted to 8.0) and mix thoroughly. Incubate in a 65 °C water bath for 60 min to lyse cells and release DNA. Add an equal volume of chloroform–isoamyl alcohol (24:1) for extraction, mix, and centrifuge at 13,000× *g* for 10 min. Carefully transfer the upper aqueous phase containing DNA to a new centrifuge tube. Add an equal volume of pre-chilled isopropanol to the aqueous phase and centrifuge at 13,000× *g* for 10 min to collect the precipitate. Wash the pellet with 70% ethanol to remove residual salts, air-dry, and dissolve in an appropriate volume of TE buffer to obtain genomic DNA. DNA quality was assessed using a NanoDrop Ultra Microvolume UV–Vis Spectrophotometer (Thermo Scientific, Waltham, MA, USA). The final A_260_/A_280_ ratio was 1.8–2.0, and the A_260_/A_230_ ratio was 2.0–2.2.

### 2.3. PCR

PCR amplification was performed using Taq DNA polymerase. Each 10 μL reaction contained 40 ng of genomic DNA, 0.5 μM of each specific forward and reverse primer, and 2× Rapid Taq Master Mix (Vazyme, Nanjing, China). The thermal cycling conditions were: 95 °C for 3 min; 30 cycles of 95 °C for 15 s, annealing for 15 s at gradient temperatures (50–63 °C) for gradient PCR or at 50–55 °C for standard PCR, and 72 °C for 15 s; with a final extension at 72 °C for 2 min. PCR products were analyzed by 2% agarose gel electrophoresis, and results were analyzed using Bio-Rad Image Lab software (version 3.0).

### 2.4. Quantitative PCR

Quantitative real-time PCR (qPCR) was performed on a CFX Duet Real-Time PCR System (Bio-Rad, Hercules, CA, USA) using 2× SYBR Green PCR Master Mix (Magen, Guangzhou, China) in a 20 μL reaction mixture containing 100 ng of genomic DNA and 1.0 μM of each specific forward and reverse primer. The thermal cycling conditions were: 95 °C for 3 min; 40 cycles of 95 °C for 15 s, 55 °C for 15 s, and 72 °C for 30 s, with fluorescence signal collection at the end of each cycle. After PCR cycling, a melting curve was generated using the default settings of the software (Bio-Rad CFX Maestro 1.1), and the threshold for Ct values was automatically determined by the software. The amplification efficiency of the primers was determined by the standard curve method using a 4-fold serial dilution series (100, 25, 6.25, 1.5625, and 0.390625 ng/μL) and calculated from the slope of the linear regression between Ct values and log-transformed concentrations. The proportion of mutant DNA was calculated using the following formula: mutant (%) = 100 × (1 − 2^−ΔΔCt^). The calculation process is consistent with conventional qRT-PCR. The control F and control R primers (which serve as internal control genes), as well as the MES F and control R primers (which serve as target genes), are used to amplify the wild-type and mutant DNA respectively. After calculating the 2^−ΔΔCt^ value, the proportion of mutant DNA is obtained by subtracting this value from 1. All qPCR assays were performed with technical replicates.

### 2.5. T7 Endonuclease I (T7EI) Cleavage Assay

Amplify a 300–1000 bp fragment containing the target site from wild-type and mutant DNA. Mix equal amounts of the two PCR products. Denature at 95 °C for 5 min to melt double strands and then slowly cool (at a rate of −2 °C/s from 95 °C to 85 °C, followed by −0.1 °C/s from 85 °C to 25 °C) to allow random reannealing and heteroduplex formation. Add 1 μL of T7EI enzyme (NEB, Ipswich, MA, USA), 2.5 μL of 10× buffer, and ddH_2_O to a final volume of 25 μL. Incubate at 37 °C for 15–30 min for digestion. Immediately analyze by 2% agarose gel electrophoresis and quantify band intensities using Bio-Rad Image Lab software. The mutant percentage was calculated as mutant (%) = 100 × (1 − √(1 − (b + c)/(a + b + c))), where a is the intensity of undigested PCR products, and b and c are the intensities of the two digested products.

## 3. Results

### 3.1. Mismatch Primer Design for MES-PCR and Comparative Evaluation with ACT-PCR

**Figure 1:**
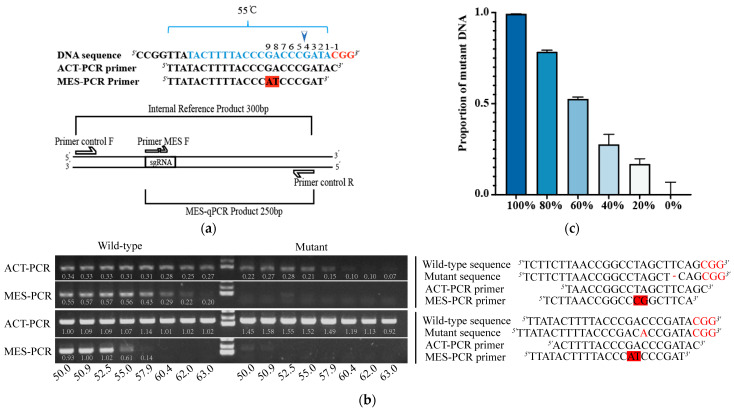
Schematic of the MES-PCR strategy and benchmark with ACT-PCR. (**a**) Primer design for mutation detection. The target DNA region is shown with the sgRNA sequence in blue and the PAM in red. Similar to ACT-PCR design, the MES-PCR assay employs two forward primers (F1, internal control; F2, mutation-specific) and a common reverse primer. The mutation-specific primer (MES F) is designed with its 3′ end at the second base upstream of the PAM and incorporates two deliberate mismatches (red background) at positions 7–8 from its 3′ end. The internal control and mutation-specific amplicons are approximately 300 bp and 250 bp in length, making them suitable for qPCR analysis. (**b**) Comparison of annealing temperature compatibility between ACT-PCR and MES-PCR. Wild-type and mutation templates were amplified using both methods across an annealing temperature gradient (50.0–63.0 °C). The corresponding DNA and primer sequences for one representative target site are shown on the right. Deletions (-), inserted bases, and the PAM sequence are shown in red, and mismatches in the primers are highlighted with a red background. Original gel images are shown in the Supplementary Materials. (**c**) Quantitative capability of MES-qPCR. PCR products from mutant and wild-type DNA were mixed at the indicated ratios (0–100% mutant DNA) and analyzed by MES-qPCR to determine the proportion of mutant DNA. The proportion of mutant DNA (mean ± SD, *n* = 3) closely matched the expected input ratio, validating the method’s accuracy in quantifying mutation frequencies.

Since Cas9 cleavage occurs 3 bp upstream of the PAM, mutations are typically introduced in the immediate vicinity of this site. Our review of multiple studies indicated that the vast majority of these mutations fall within a window of 2–7 bp upstream of the PAM [15,16,17,18,19]. Accordingly, we anchored the 3′ end of our identification primer at the second base upstream of the PAM. To enhance specificity, two mismatched bases were deliberately introduced at positions 7–8 from the primer’s 3′ end (corresponding to 8–9 bp upstream of the PAM), such that the primer’s annealing temperature is approximately 55 °C (Figure 1a). In contrast, the ACT-PCR primer is designed with its 3′ end at the first base of the PAM without any mismatch. A common reverse primer was placed approximately 250 bp downstream of the PAM, and an internal control forward primer was designed approximately 50 bp upstream of the PAM (Figure 1a). To facilitate the detection of multiple different mutations in a single PCR run (i.e., using the same thermal cycling program on the same PCR block), it is recommended to use similar amplicon lengths and similar annealing temperatures. The spatial relationship between these primers is illustrated in Figure 1a.

To assess the sensitivity of the designed primers against mutations, we conducted annealing temperature gradient PCR on two known point-mutated DNA fragments (previously obtained by our laboratory that had already been sequenced), comparing the performance of MES-PCR primers with conventional ACT-PCR primers. MES-PCR primers efficiently amplified the two wild-type templates at lower annealing temperatures, while showing negligible amplification of mutant fragments even at the minimum temperature tested (Figure 1b and Figure S1). In contrast, ACT-PCR primers could only discriminate one of the two mutated fragments at temperatures above 60 °C and failed to distinguish the other mutation below 63 °C, which was located farther from the PAM (Figure 1b). Meanwhile, we tested the effects of various mismatches introduced into the primers on PCR amplification and found that they had little to no impact on the final results (Figure S1). Therefore, when introducing mismatches, only the specificity of the primers needs to be considered. These findings demonstrate that MES-PCR provides a reliable and temperature-tolerant approach for mutation detection. Its effectiveness does not rely on stringent annealing temperature control; therefore, the annealing temperature can be appropriately reduced, or the number of cycles can be increased in experiments to improve the amplification efficiency of the internal control wild-type fragment without affecting mutant detection, thereby simplifying the genotyping workflow.

In cases in which quantifying the proportion of edited alleles within a sample is required, we validated the quantitative capability of MES-PCR primers on a qPCR platform. Wild-type and mutated PCR products (from Figure 1b) were mixed at predetermined ratios and subjected to MES-qPCR analysis. The results demonstrated that the proportion of mutations calculated by MES-qPCR closely correlated with the expected mixing ratios (Figure 1c), confirming its accuracy in detecting mutation frequencies. Therefore, MES-qPCR can reliably determine the proportion of mutations in DNA samples. Furthermore, since heterozygotes in the T1 generation theoretically contain 50% mutant DNA, this approach also provides an efficient means for screening T1 heterozygous individuals.

Collectively, these results demonstrate that MES-PCR outperforms ACT-PCR in discriminating wild-type from mutant alleles. Its key advantage lies in eliminating the dependency on stringent annealing temperature control, thereby simplifying the workflow by removing the need for temperature gradient optimization; a uniform annealing temperature of 55 °C is typically effective. Furthermore, when integrated with quantitative PCR (MES-qPCR), the method enables accurate quantification of mutation frequencies within DNA samples, providing a reliable tool for assessing sgRNA editing efficiency and screening heterozygous individuals.

### 3.2. Rapid and Cost-Effective Screening of sgRNA Efficiency by MES-qPCR

**Figure 2:**
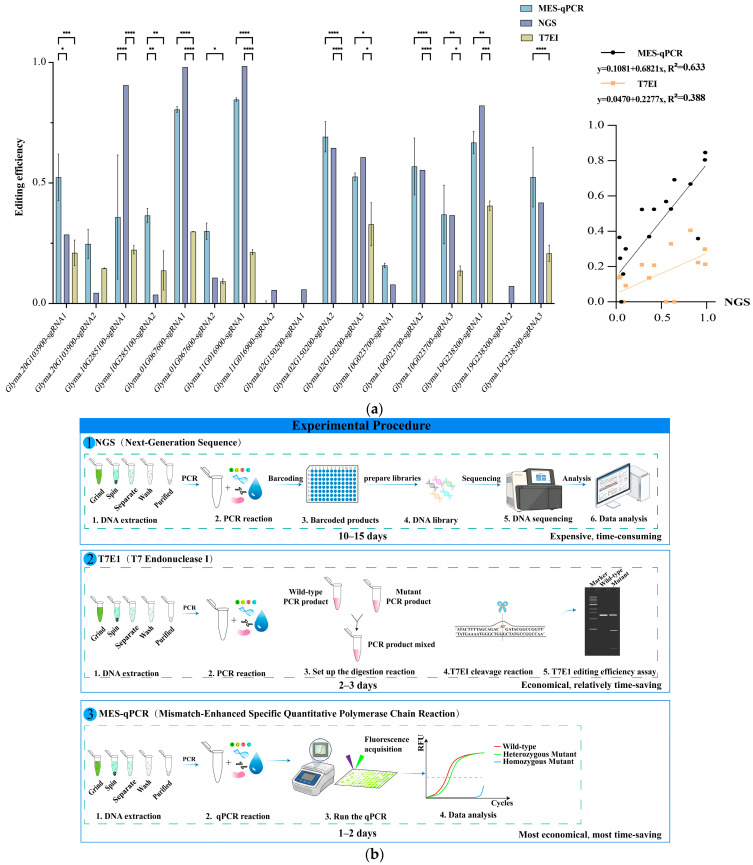
Comparative benchmarking of three methods for assessing sgRNA editing efficiency in soybean hairy roots. (**a**) Comparison of editing efficiency detection methods. The editing efficiencies of 17 sgRNAs targeting 7 soybean genes were evaluated in parallel using MES-qPCR (mean ± SD, *n* = 3), NGS and T7EI assay (mean ± SD, *n* = 2). Original T7EI assay gel images are shown in the Supplementary Materials. Two-way ANOVA was performed between groups, with significance indicated as follows: **** *p* < 0.0001, *** *p* < 0.0005, ** *p* < 0.005, * *p* < 0.05. The right panel presents the results of the regression analysis. (**b**) Workflow comparison of three methods. Schematics illustrate the procedural steps and approximate hands-on time required for sgRNA efficiency assessment using NGS, T7EI, and MES-qPCR, highlighting differences in cost, time, and operational complexity.

For plant species that are challenging to stably transform—such as soybean, which involves a lengthy and low-efficiency process—it is crucial to pre-screen and validate highly efficient sgRNAs before committing to stable transformation. To this end, we designed multiple sgRNAs targeting the soybean genome and employed the *Agrobacterium rhizogenes* strain K599-mediated hairy root transformation system for rapid efficiency evaluation [20]. Following transformation, genomic DNA was extracted from the hairy roots, and the editing efficiencies of the sgRNAs were assessed using three complementary methods: T7EI assay, Hi-TOM (The raw data are provided in the Supplementary Materials.) [7], and MES-qPCR (Figure 2a). Using NGS results as the reference standard, we observed that the T7EI results consistently underestimated editing frequencies. While MES-qPCR results for some sgRNAs (e.g., gene1-sgRNA2, gene2-sgRNA1, gene2-sgRNA2) showed notable deviations from NGS data, its overall accuracy (mean absolute error = 0.15) remained substantially higher than that of the T7EI assay (mean absolute error = 0.27). Meanwhile, regression analysis showed that MES-qPCR had a higher correlation with NGS (R^2^ = 0.633) than with T7EI (R^2^ = 0.388) (Figure 2a).

The T7EI assay is widely adopted for initial screening owing to its simplicity and low cost. However, it is semi-quantitative at best, frequently underestimating editing frequencies and showing variable sensitivity depending on mismatch positions, which can lead to false negatives or ambiguous results [5]. In contrast, NGS is considered the gold standard for precise quantification of editing efficiency and for characterizing the full spectrum of editing outcomes, including indel distribution. Nevertheless, NGS requires expensive instrumentation or service fees, substantial bioinformatics expertise, and longer turnaround times, rendering it less practical for large-scale sgRNA screening prior to stable transformation [7]. MES-qPCR presents a balanced alternative: it retains the simplicity of PCR-based detection while achieving relatively high accuracy. Thus, it provides a rapid, cost-effective, and high-throughput-compatible method for assessing sgRNA efficiency, making it particularly suitable for routine screening within plant transformation and editing pipelines.

### 3.3. Cross-Generational Screening of Heterozygous and Homozygous Mutants in Plants Using MES-PCR

**Figure 3:**
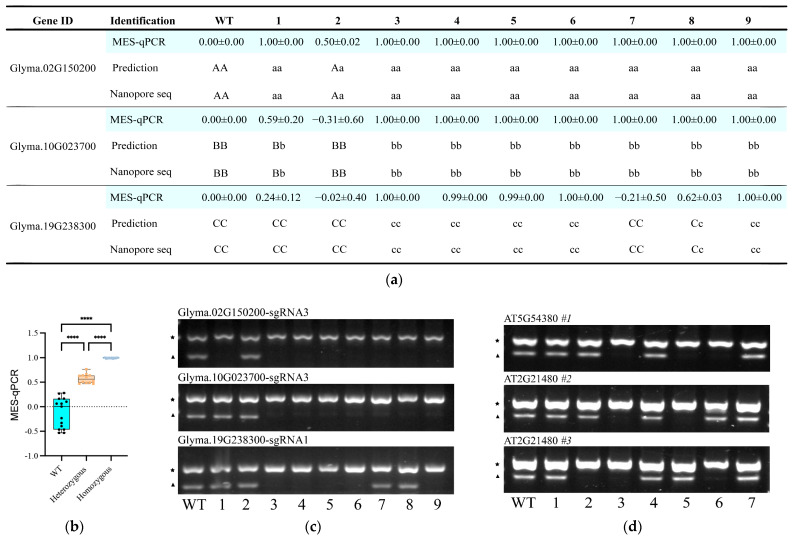
Accuracy of MES-qPCR in genotyping of soybean and *Arabidopsis thaliana*. (**a**) Comparison of the MES-qPCR results and the sequencing results in soybean. MES-qPCR was performed in three technical replicates, and data are presented as mean ± SD (*n* = 3). Uppercase letters (A/B/C) indicate wild-type alleles; lowercase (a/b/c) indicate mutant alleles. (**b**) Distribution of MES-qPCR data for the three genotypes. One-way ANOVA was performed between groups, with significance indicated as follows: **** *p* < 0.0001. (**c**) Genotyping 9 soybean seedlings in Figure 3a by MES-PCR. The five-pointed star (★) and the triangle (▲) represent the internal control fragment and the MES-PCR fragment, respectively. Original gel images are shown in the Supplementary Materials. (**d**) Genotyping of 7 T_2_ seedlings from each of the three T_1_ heterozygous knockout lines shown in Figure S2a by MES-PCR. The five-pointed star (★) and the triangle (▲) represent the internal control fragment and the MES-PCR fragment, respectively. Original gel images are shown in the Supplementary Materials.

To validate MES-qPCR for genotyping, we first applied it to screen T2 generation knockout lines for three soybean genes. A total of nine lines were analyzed, and the mutation status determined by MES-qPCR was consistent with the results from nanopore sequencing (Figure 3a). Linear regression of MES-qPCR against the actual genotype scores (0 for wild type, 0.5 for heterozygote, and 1 for homozygote) demonstrated excellent concordance (R^2^ > 0.96). We then compiled the MES-qPCR data for all mutation types and observed that the result distributions for the three genotypes exhibited virtually no overlap (Figure 3b). On this basis, we suggest classifying lines with MES-qPCR values >0.85 as homozygous mutants (including compound heterozygotes), values between 0.45 and 0.85 as heterozygotes, and values <0.45 as wild type. We also used MES-PCR to identify these nine T2 knockout mutations for each of the three genes, respectively. For ease of observation, we mixed the PCR products of the internal control fragment and the MES-PCR fragment and loaded them into the same gel well. The results showed that individuals 1, 3, 4, 5, 6, 7, 8 and 9 carried homozygous mutations in Glyma.02G150200-sgRNA3; individuals 3, 4, 5, 6, 7, 8 and 9 carried homozygous mutations in Glyma.10G023700-sgRNA3; and individuals 3, 4, 5, 6 and 9 carried homozygous mutations in Glyma.19G238300-sgRNA1. Consolidating these results, lines 3, 4, 5, 6 and 9 were readily identified as triple homozygous mutations (Figure 3c). We then performed a preliminary screening by MES-qPCR on 43 T_0_ knockout lines of Glyma.02G150100. Six lines exhibiting elevated MES-qPCR signals were subjected to nanopore sequencing—given that T_0_ individuals can be chimeric and thus the mutation ratios may deviate from 50%—and all proved to be heterozygous (Figure S3a). We then examined nine progeny lines from line 16 using MES-PCR and found that, with the exception of lines 2 and 3, all were homozygous mutants (Figure S3b), a result that agreed with the sequencing data (Figure S3c).

We next extended the application of MES-qPCR to *Arabidopsis thaliana* genes. Single sgRNA knockout vectors for three target genes were constructed using the pHEE401e vector system [21] and were transformed into Col-0 plants. MES-qPCR was then employed to identify mutations in the T1 generation (Figure S2a). Lines with mutation probability exceeding 0.5, as estimated by MES-qPCR, were selected for confirmation by Sanger sequencing (Figure S2c). This result further confirmed that lines with probabilities near 1 were homozygous, while those near 0.5 were heterozygous.

This screening strategy aligns with the practical realities of plant gene editing: T1 populations frequently comprise a mixture of heterozygotes, necessitating quantitative methods like MES-qPCR for accurate genotyping. In contrast, homozygous lines in the T2 generation can be rapidly identified using MES-PCR. Following this workflow, we confirmed that T2 lines 3, 5, and 6 of AT5G54380 #1 (derived from T1 #5), and T2 lines 3 and 5 of AT2G21480 #2 (derived from T1 #2), and T2 lines 2, 3, and 6 of AT2G21480 #3 (derived from T1 #7) were homozygous mutants (Figure 3d). It is noteworthy that using highly efficient sgRNA with the egg cell-specific EC1.2 promoter driving Cas9 expression, which can directly generate homozygous T1 mutations [21] (Figure S2). Consequently, MES-qPCR establishes a versatile and efficient framework for the identification of edited mutants across different plant generations, from the quantitative analysis of segregating T1 populations to the confirmation of stable homozygotes in subsequent generations.

## 4. Discussion

In this study, we developed a highly cost-effective and reliable method for identifying single-base mutations generated by the Cas9 editing system via rationally optimized primer design. MES-PCR is conceptually analogous to ACT-PCR but imposes less stringent experimental conditions, enabling clear discrimination between mutations and wild type even at low and fixed annealing temperatures. Furthermore, it can identify mutations located at greater distances from the PAM sequence. Combined with quantitative PCR, the method allows calculation of sgRNA efficiency and demonstrates robust screening capability for heterozygous mutations.

The introduction of two mismatches at positions 7–8 from the 3′ end of the primer may exert negative impacts on PCR performance. In particular, amplification efficiency may slightly decrease, as reflected by increased Ct values or lower amplicon yield; notably, this effect is relatively minor when the mismatches are weak (e.g., G-T) [22]. In contrast, strongly destabilizing mismatches such as T-T or C-C can compromise duplex stability and potentially lead to amplification failure, although we have not observed such cases with wild-type DNA in our experiments (Figure S2a). If this issue occurs, alternative mismatched combinations can be considered when designing MES-PCR primers.

We observed occasional negative values in MES-qPCR quantitation, and statistical analysis revealed that they predominantly occurred in the wild-type data, accounting for more than 50% (Figure 3b). Under ideal circumstances, the calculated mutation ratios should range from 0 to 1. Out-of-range values likely arise from technical artifacts, including: (1) DNA sample impurities that impair amplification efficiency (particularly in large-scale extraction workflow) or (2) pipetting inaccuracies during PCR preparation. In such cases, we either classified the samples directly as wild type or repeated the entire detection procedure to ensure reliable genotyping.

Mutations induced by the Cas9 system encompass not only single-nucleotide mutations but also a substantial fraction of multi-base deletions [15,16]. Such deletions also occur within the sequence targeted by our primers, making MES-PCR broadly applicable to most Cas9-induced mutations. However, rare exceptions may occur—for instance, when an identical base is inserted precisely 2 bp upstream of the PAM or when the induced mutation matches the artificial mismatches engineered into the primer—such scenarios may prevent discrimination between wild-type and mutant alleles. Fortunately, the occurrence rate of these exceptional events is extremely low, and they exert negligible influence.

Overall, MES-PCR offers a simpler strategy for screening mutations generated by the Cas9 editing system. Nonetheless, this method inherits certain limitations of qPCR, as any factor that compromises PCR amplification may lead to result misinterpretation. External factors mainly include the purity of the DNA sample and the accuracy of pipetting, whereas internal factors primarily involve variable binding efficiencies of distinct mutation types to the same primer. In future work, further optimization of primer design can be explored to minimize PCR-associated errors. Furthermore, we aim to optimize primer design and the PCR system to reduce the need for qPCR in heterozygote identification.

## 5. Conclusions

MES-PCR is a primer-design-based mutation screening method. Its core workflow involves introducing two mismatched bases at positions 7–8 from the 3′ end of the forward discrimination primer, whereby wild-type templates are efficiently amplified while mutant templates show little to no amplification. When combined with qPCR (MES-qPCR), this system also enables quantification of mutation proportions, supporting reliable evaluation of sgRNA editing efficiency and screening of heterozygous mutations. The main advantages of this method are its independence from stringent annealing temperature optimization, its lack of reliance on restriction enzyme sites or expensive instruments, its experimental simplicity and low cost, and its superior detection accuracy compared with T7EI—all of which make it an effective preliminary screening tool.

## Figures and Tables

**Table 1:** Comparison of present methods for point mutation identification.

Method	Brief Workflow	Time Required	Cost	Operational Complexity	Result Accuracy
ARMS-PCR [1,9]	Design allele-specific primers (with the 3′ end matching the target mutation), perform PCR, and detect amplified products by gel electrophoresis.	2–3 h	Low	Moderate (due to the requirement for allele-specific primer design)	Moderate (limited to known mutations, possible false positives)
ACT-PCR [2]	Determine a critical annealing temperature by gradient PCR; mutant amplicons yield no detectable band on gel.	3–4 h	Low	Moderate (requires precise temperature optimization; ineffective for heterozygous mutation detection)	Moderate (cannot distinguish heterozygotes; sensitive to 1 bp indels)
PCR/RE [3,10]	Amplify target region by PCR, digest with restriction enzyme, and analyze fragments by gel electrophoresis (requires the mutation to disrupt a restriction site).	3–4 h	Low	Moderate (requires a suitable restriction site; involves a multiple-step procedure)	High (dependent on restriction site availability; underestimates efficiency)
PCR/RNP [4]	Assemble the Cas9 protein and sgRNA into a ribonucleoprotein complex in vitro, incubate with PCR products, and detect specific cleavage.	3–5 h	Moderate	Relatively complex (requires in vitro assembly of Cas9-sgRNA complexes)	High (high sensitivity but requires RNP preparation)
T7EI [3,5,11]	Denature and re-anneal PCR products to form heteroduplexes, digest with T7 endonuclease I, and analyze the cleavage pattern by gel electrophoresis.	4–5 h	Low	Moderate (involves enzymatic digestion and electrophoresis steps)	Low (underestimates editing efficiency; insensitive to small indels)
HRMA [6,11]	Perform real-time PCR followed by high-resolution melting curve analysis to characterize melting profiles.	2–3 h	High (requires a HRM instrument)	Simple (process is largely automated)	Moderate (limited sensitivity for G-C/A-T variations)
Sanger sequencing [12,13]	Amplify target region by PCR, purify product, perform cycle sequencing, and perform capillary electrophoresis for base calling.	1–2 days	Relatively high	Simple (sequencing service available)	High (signals easily masked when allele frequency <10%)
NGS [11] (e.g., Hi-TOM [7])	Design multiplex PCR primers with barcodes, construct a library, perform high-throughput sequencing, and perform bioinformatic analysis to determine mutation events.	3–5 days	Moderate to high (per-sample cost can be reduced via multiplexing)	Complex (requires sequencing platform and data processing capability)	High (can detect low-frequency mutations; high throughput)
Nanopore sequencing [8,14]	PCR amplify target region, perform library preparation, load onto flow cell, perform real-time sequencing, analyze with bioinformatics tools.	1–2 days	Low to medium	Simple (sequencing service available)	Moderate to high (high throughput; point mutation detection limited by native error rate, can be improved via computational correction)

## Data Availability

Other relevant data supporting the conclusions of this article are presented within the article or Supplementary Materials.

## Supplementary Materials

The following supporting information can be downloaded at https://www.mdpi.com/article/10.3390/biology15131089/s1: supplementary data1: Figure S1. Effects of various mismatches introduced into the primers on PCR amplification; Figure S2. Genotyping of *Arabidopsis* T1 gene-edited lines by MES-qPCR and Sanger sequencing; Figure S3. Genotyping of soybean T0 and T1 gene-edited lines; supplementary data2: primer sequences and the raw data corresponding to all figures. Original Images for Gels.zip. All raw sequencing data can be downloaded at https://doi.org/10.5281/zenodo.20524705.

## Abbreviations

The following abbreviations are used in this manuscript:

MES-PCR	Mismatch-enhanced specific polymerase chain reaction
MES-qPCR	Mismatch-enhanced specific real-time quantitative polymerase chain reaction
T7EI	T7 endonuclease I
NGS	Next-generation sequencing
PAM	Protospacer adjacent motif
