# Supplementary material for: Mismatch-Enhanced Specific PCR (MES-PCR): A Rapid and Cost-Effective Method for Screening CRISPR/Cas9-Induced Mutations
Source: Biology (Basel). 2026 Jul 7;15(13):1089. doi: 10.3390/biology15131089 (PMC13360154; doi:10.3390/biology15131089)
Supplement: Supplementary file 1 [file biology-15-01089-s001.zip › supplementary data1.pdf]

Supplementary Material

Mismatch-Enhanced Specific PCR (MES-PCR): A Rapid and Cost-Effective Method for Screening CRISPR/Cas9-Induced Mutations

Peng Tian[1,2,†], Bengang Yao[1,2,†], Wenjing Lin[1,2], Maoting Yuan[1,2], Shuran Li[1,2], Yuzhu Qin[1,2], Shuang Chen[1,2], Tao Lai[3], Zhenbiao Yang[2,3], Wenwei Lin[2*], Xiang Zhou[3*]

1 Fujian Provincial Key Laboratory of Haixia Applied Plant Systems Biology, College of Life Science, Fujian Agriculture and Forestry University, Fuzhou 350002, China

2 Horticultural Plant Biology and Metabolomics Center, Haixia Institute of Science and Technology, Fujian Agriculture and Forestry University, Fuzhou 350002, China

3 State Key Laboratory of Quantitative Synthetic Biology, Shenzhen Institute of Synthetic Biology, Shenzhen Institutes of Advanced Technology, Chinese Academy of Sciences, Shenzhen 518107, China

† These authors contributed equally to this work

* Correspondence: Xiang Zhou: x.zhou3@siat.ac.cn, Wenwei Lin: wwlin@fafu.edu.cn

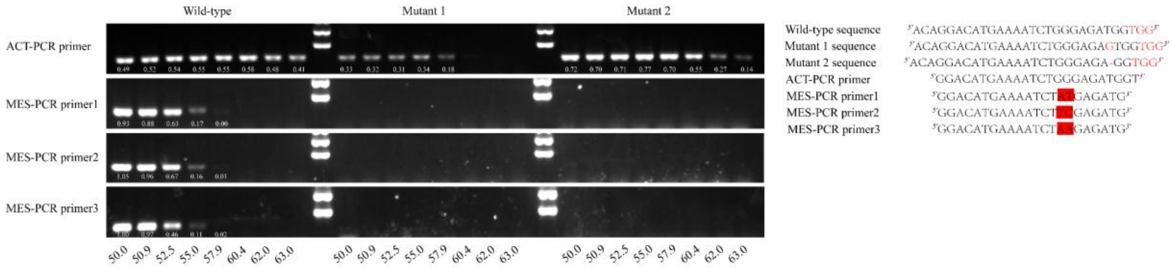

Figure S1. Effects of various mismatches introduced into the primers on PCR amplification. Wild-type and two mutant templates were subjected to gradient PCR (50.0–63.0 °C) with ACT-PCR primers and MES-PCR primers, respectively. A total of three primers were designed for MES-PCR, each incorporating mismatches that exhibit different binding efficiencies. The corresponding DNA and primer sequences are presented on the right. Deletions (-), inserted bases, and the PAM sequence are shown in red, and mismatches in the primers are highlighted with a red background.

Gene	Col-0	1	2	3	4	5	6	7	8	9	10	11	12	13	14	15
At5g39000	0	-0.28	0.99	0.99	0.15	0.13	0.04	0.1	0.3	0.98	1	0.14	0.99	0.43	0.99	0.99
At5g54380	0	0.24	0.13	0.06	0.37	0.77	0.34	0.11	-0.11	0.11	0.22	-0.18	0.38	0.42		
At2g21480	0	0.99	0.52	0.14	-0.03	0.36	-0.02	0.47	0.24	-0.53	-0.29	0.99	-0.34	0.19	-0.34	

(a)

At5g39000	AC CCGATACGG	At5g54380	ATCTC TTGTGG
2	ACTCCGATACGG<br>ACACCGATACGG	5	ATCTCATTGTGG<br>ATCTC TTGTGG
3	ACTCCGATACGG<br>AC -CGATACGG		
9	ACTCCGATACGG<br>ACTCCGATACGG	At2g21480	TAGCTT CAGCGG
10	ACACCGATACGG<br>ACACCGATACGG	1	TAGCTTACAGCGG<br>TAGCTTTCAGCGG
12	ACTCCGATACGG<br>ACTCCGATACGG	2	TAGCTTTCAGCGG<br>TAGCTT CAGCGG
14	ACACCGATACGG<br>ACACCGATACGG	7	TAGCTT -AGCGG<br>TAGCTT CAGCGG
15	ACTCCGATACGG<br>ACACCGATACGG	11	TAGCT- CAGCGG<br>TAGCT- CAGCGG

(b)

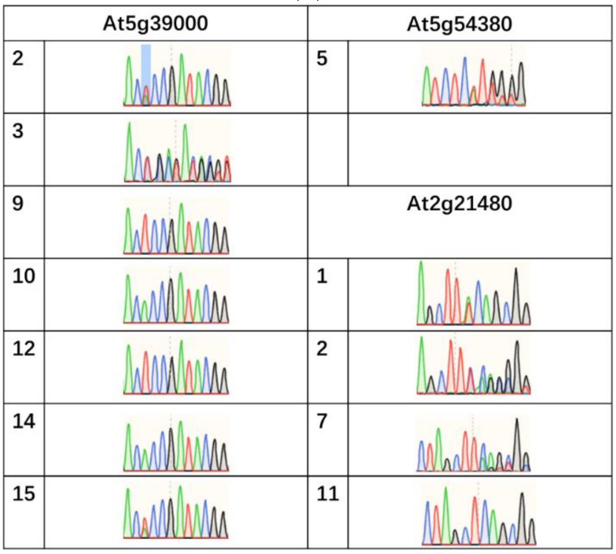

(c)

Figure S2. Genotyping of *Arabidopsis* T1 gene-edited lines by MES-qPCR and Sanger sequencing. (a) Mutation probability for each T1 line as determined by MES-qPCR. Lines with a dark background indicate homozygous mutations, those with a light background indicate heterozygotes, and those with a white background indicate the wild type. (b) Sanger sequencing results corresponding to the lines shown in (a). The sequences from two chromosomes are shown, with insertions highlighted in red, deletions denoted by a dash ("-"), and red letters indicating the PAM sequence. (c) Representative Sanger sequencing chromatograms corresponding to the sequences displayed in (b).

Gene ID	Identification	WT	1	2	3	4	5	6	7	8	9
Glyma.02G150100	MES-qPCR	0.00±0.00	0.60±0.24	0.06±0.25	0.28±0.19	0.25±0.18	0.24±0.10	0.06±0.15	0.14±0.13	0.32±0.15	0.50±0.08
	Prediction	AA	Aa	AA	AA	AA	AA	AA	AA	AA	Aa
	Nanopore seq	AA	Aa	-	-	-	-	-	-	-	Aa
			**10**	**11**	**12**	**13**	**14**	**15**	**16**	**17**	**18**	**19**
	MES-qPCR		0.25±0.06	0.14±0.04	0.16±0.10	0.13±0.09	0.19±0.11	0.19±0.00	0.80±0.07	−0.26±0.29	−0.01±0.13	−0.26±0.29
	Prediction		AA	AA	AA	AA	AA	AA	Aa	AA	AA	AA
	Nanopore seq		-	-	-	-	-	-	Aa	-	-	-
			**20**	**21**	**22**	**23**	**24**	**25**	**26**	**27**	**28**	**29**
	MES-qPCR		0.28±0.02	0.71±0.26	0.39±0.11	0.24±0.09	0.17±0.09	0.38±0.16	0.14±0.25	0.02±0.03	0.25±0.32	0.14±0.07
	Prediction		AA	AA	AA	AA	AA	Aa	AA	AA	AA	AA
	Nanopore seq		-	-	-	-	-	Aa	-	-	-	-
			**30**	**31**	**32**	**33**	**34**	**35**	**36**	**37**	**38**	**39**
	MES-qPCR		0.18±0.30	0.25±0.25	0.28±0.10	0.19±0.14	0.40±0.35	0.35±0.19	0.41±0.14	0.44±0.23	−0.33±0.30	0.22±0.12
	Prediction		AA	AA	AA	AA	AA	AA	Aa	AA	AA	AA
	Nanopore seq		-	-	-	-	-	-	Aa	-	-	-
			**40**	**41**	**42**	**43**						
	MES-qPCR		0.06±0.11	0.05±0.09	0.43±0.12	−0.38±0.34						
	Prediction		AA	AA	Aa	AA						
	Nanopore seq		-	-	Aa	-						

(a)

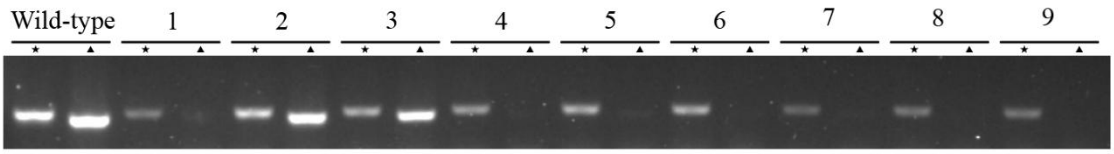

Glyma.02G150100

(b)

Glyma.02G150100: ACGGTAGGGCTGG	
1: ACGGTA - - GCTGG	
2: ACGGTAGGGCTGG	
3: ACGGTAGGGCTGG	
4: ACGGTA - - GCTGG	
5: ACGGTA - - GCTGG	
6: ACGGTAG - GCTGG	
7: ACGGTA - - GCTGG	
8: ACGGTAG - GCTGG	
9: ACGGTA - - GCTGG	

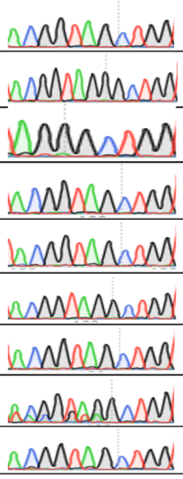

(c)

Figure S3. Genotyping of soybean T0 and T1 gene-edited lines. (a) Comparison of MES-qPCR results with sequencing results in soybean T0 generation. MES-qPCR was performed in three technical replicates, and data are presented as mean ± SD (n = 3); the dash (−) indicates that sequencing was not performed. Uppercase letters (AA) indicate wild-type alleles; lowercase (aa) indicate mutant; heterozygous are shown as one of each. (b) Genotyping of 9 soybean T1 lines derived from line 16 in (a) by MES-PCR. The five-pointed star (★) and triangle (▲) represent the internal control fragment and the MES-PCR fragment, respectively. (c) Representative Sanger sequencing chromatograms corresponding to the sequences shown in (b). Deletions denoted by a red dash ("-").
